# Failure mechanical properties of lumbar intervertebral disc under high loading rate

**DOI:** 10.1186/s13018-023-04424-x

**Published:** 2024-01-03

**Authors:** Qing Liu, Xiao-Feng Liang, Ai-Guo Wang, Ying Liu, Tong-Ju Jia, Kun Li, Chun-Qiu Zhang

**Affiliations:** 1 Tianjin Key Laboratory for Advanced Mechatronic System Design and Intelligent Control, School of Mechanical Engineering, Tianjin University of Technology, Tianjin, 300384 People’s Republic of China; 2 National Demonstration Center for Experimental Mechanical and Electrical Engineering Education, Tianjin University of Technology, Tianjin, 300354 People’s Republic of China; 3 Department of Mechanics, Tianjin University, Tianjin, 300354 People’s Republic of China; 4 Tianjin Key Laboratory of Nonlinear Dynamics and Control, Tianjin, 300354 People’s Republic of China; 5 Affiliated Hospital of Tianjin Academia Sinica, Tianjin, 300120 People’s Republic of China; 6 Tianjin Key Laboratory of Film Electronic and Communication Device, Tianjin University of Technology, Tianjin, 300384 People’s Republic of China

**Keywords:** Lumbar disc herniation, Failure mechanism, High loading rate, Constitutive model, Stress–strain curves, Internal displacement distribution

## Abstract

**Background:**

Lumbar disc herniation (LDH) is the main clinical cause of low back pain. The pathogenesis of lumbar disc herniation is still uncertain, while it is often accompanied by disc rupture. In order to explore relationship between loading rate and failure mechanics that may lead to lumbar disc herniation, the failure mechanical properties of the intervertebral disc under high rates of loading were analyzed.

**Method:**

Bend the lumbar motion segment of a healthy sheep by 5° and compress it to the ultimate strength point at a strain rate of 0.008/s, making a damaged sample. Within the normal strain range, the sample is subjected to quasi-static loading and high loading rate at different strain rates.

**Results:**

For healthy samples, the stress–strain curve appears collapsed only at high rates of compression; for damaged samples, the stress–strain curves collapse both at quasi-static and high-rate compression. For damaged samples, the strengthening stage becomes significantly shorter as the strain rate increases, indicating that its ability to prevent the destruction is significantly reduced. For damaged intervertebral disc, when subjected to quasi-static or high rates loading until failure, the phenomenon of nucleus pulposus (NP) prolapse occurs, indicating the occurrence of herniation. When subjected to quasi-static loading, the AF moves away from the NP, and inner AF has the greatest displacement; when subjected to high rates loading, the AF moves closer to the NP, and outer AF has the greatest displacement. The Zhu–Wang–Tang (ZWT) nonlinear viscoelastic constitutive model was used to describe the mechanical behavior of the intervertebral disc, and the fitting results were in good agreement with the experimental curve.

**Conclusion:**

Experimental results show that, both damage and strain rate have a significant effect on the mechanical behavior of the disc fracture. The research work in this article has important theoretical guiding significance for preventing LDH in daily life.

## Introduction

Lumbar disc herniation (LDH) is the main clinical cause of low back pain, which is usually caused by the prolapse of the nucleus pulposus through ruptured annulus fibrosus [1]. The pathogenesis of lumbar disc herniation is very complex, and its principle is still uncertain, but the occurrence of disc rupture often accompanies it [2]. Normally, it is caused by the accumulation of long-term mechanical load on the lumbar intervertebral disc and the sudden overload injury [3]. In general, there are two types of structural failures exhibited in intervertebral disc: endplate fracture and annulus fibrosus rupture [4]. These fissures do not seem to cause pain in the early stages, but they have been shown to aggravate the degeneration of the intervertebral disc [5]. Wade et al. experimented with different loading positions for sheep lumbar vertebrae containing preexisting defects in the central dorsal annulus, and by comparing the results of scanning the intervertebral discs in ultrahigh-field MRI (magnetic resonance imaging, 11.7T) before and after the test, they found that they contained a greater degree of preexisting damage and were more prone to herniated discs [6]. Loading rate and posture are key factors in the herniation process [7], previous studies have shown that vertical compression often leads to endplate fractures or vertebral body injuries, and flexion increases the likelihood of annular failure, while the accuracy of the failure depends on the loading rate [8]. For instance, Wade et al. carried out loading experiments with sheep lumbar spine at different speeds and observed the microstructure of the damaged intervertebral disc. They hypothesized the location of the initiation of annulus fibrous rupture and the mode of diffusion by comparing the categories of disc damage caused by different loads, at the same time, it also reflects that the loading rate also affects the rupture of the intervertebral disc [9]. LI et al. carried out low, medium and high-rate loading of the intervertebral disc, and after observing the mechanical differences in intervertebral disc rupture, they pointed out that the yield phenomenon will occur at these three speeds, but the rupture phenomenon only occurs at medium and high-speed loading [10]. Although many studies have been performed on how the intervertebral disc fails under various conditions, the mechanism of disc failure and herniation is still not fully understood.

Lumbar discs are viscoelastic in nature and can therefore exhibit different mechanical responses to different strain rates [11]. For instance, Wade et al. conducted compression rupture experiments on sheep lumbar spine at high loading speed and found motion segments subjected to a “surprise” loading rate are likely to fail via some form of annular rupture. Failure under such sudden loading occurs mostly via rupture of the annular-endplate junction and is thought to arise from a rate-induced mechanostructural imbalance between the annulus and the endplate [12]. In daily life, the human body will inevitably encounter falls, slips, and other conditions. In order to explore the potential pathogenesis of the above conditions that may lead to lumbar disc herniation, in 4 recent years, some scholars have studied the mechanical behavior of lumbar intervertebral discs at high loading rates [13, 14], the above studies indicate that loading rate has an influence on disc failure mode, however, differences in experimental equipment, loading protocol, and specimens result in a lack of comparability. Therefore, the relationship between loading rate and failure mechanics is not yet well defined.

The physically realistic constitutive representation is considered as an important method to deeply understand the origin of the deformation-induced failure mechanisms affecting the IVD function [15]. In recent years, a lot of fully three-dimensional AF model have been developed to predict the regional anisotropic multiaxial damage of the IVD with the finite element method [16, 17], and using 3D printing technology to construct an intervertebral disc model, the model provides a convenient experimental platform for evaluating normal and pathological disc states and assessing the biomechanics of potential therapeutic interventions [18, 19]. However, a complete constitutive model of IVD is not established above studies. The ZWT nonlinear viscoelastic constitutive model is composed of a nonlinear spring and two Maxwell elements in parallel, and a series of existing experimental studies have shown that this model can be used to satisfactorily describe the nonlinear viscoelastic constitutive behavior of various polymers within the strain rates range from 10^−4^/s to 10^3^/s. For example, Jiang et al. satisfactorily described the behavior of ethylene propylene diene monomer (EPDM) in 27% strain under low strain rates of 0.00025 s^−1^, 0.025 s^−1^ and high strain rates in the range of 1300 s^−1^, 2100s^−1^ using the improved ZWT nonlinear viscoelastic constitutive model [20]. Luo et al. performed a numerical simulation of the performance of epoxy resin with a split Hopkinson pressure bar (SHPB), and the simulated stress–strain curve was in good agreement with the experimental results under quasi-static compression of 1 × 10^−4^ s^−1^, 1 × 10^−3^ s^−1^, and 1 × 10^−4^ s^−1^, and a high strain rate of 650 s^−1^, 1050 s^−1^, and 1600 s^−1^. The results show that the ZWT constitutive model can better simulate the stress–strain relationship of epoxy resin in the 8% strain range and at different strain rate [21]. Zhang et al. established the dynamic constitutive model of desert sand concrete at room temperature on the basis of the ZWT constitutive model, which can better predict the stress–strain curve in the 1.25% strain range under the dynamic compression test with strain rate of 1.45 × 10^−6^ s^−1^, 1.45 × 10^−5^ s^−1^, and 1.45 × 10^−4^ s^−1^ [22]. In this article, the ZWT constitutive model is used to quantitatively describe the viscoelastic mechanical behavior of lumbar disc at high strain rate, and the physical meaning of the model parameters is thoroughly analyzed.

## Materials and methods

### Sample preparation

**Fig. 1:**
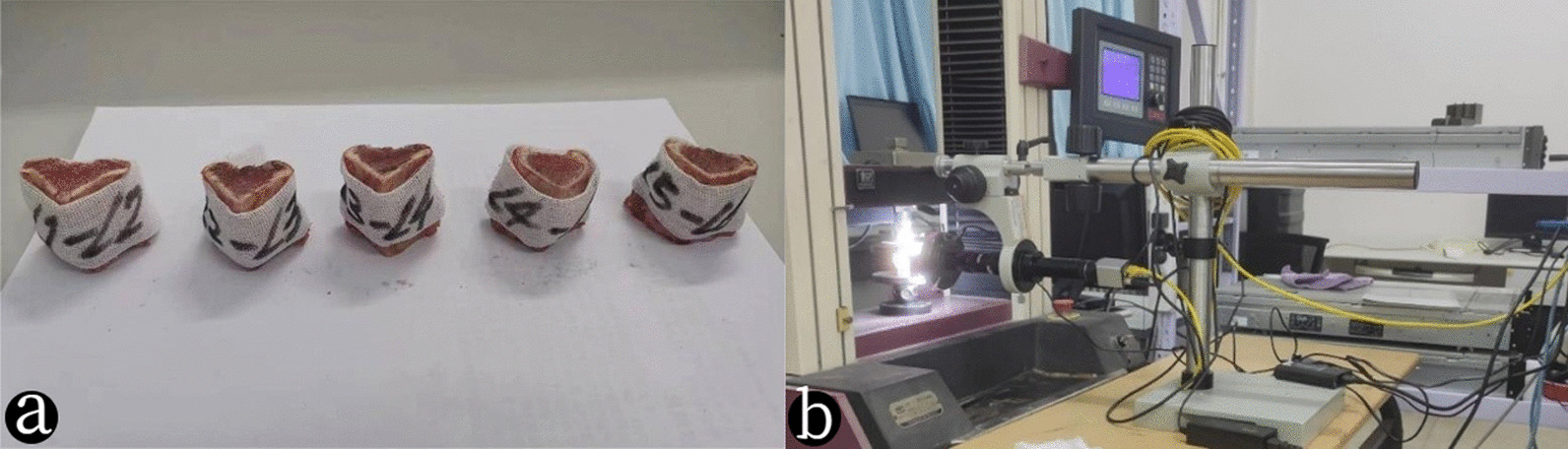
**a** Experimental sample. **b** Experimental apparatus. WDW-10 microcomputer control electronic universal testing machine is used in the quasi-static loading test, with loading range from 0 to10 KN and loading speed between 0.1 and 200 mm/min. A digital image relevant technique is used to test the internal displacement. And having largest magnification of 300×, the CCD camera is adopted with a maximum acquisition accuracy of 1376 × 1035

The samples are taken from the lumbar vertebrae of 30 adult sheep which died within 4–6 h, anterior and posterior ligaments, spinous processes, and transverse joints on the lumbar are removed, and all the elements and ligaments in the posterior part are removed along the coronal plane of the line of the diameter of the vertebral foramen (Fig. 1a). A portion of the vertebral body is left on each side of the upper and lower discs, and the upper vertebrae body is ground into a 5° inclined plane. And to better maintain the viscoelasticity of the disc, the samples are wrapped in wet gauze.

### Experimental equipment and method

WDW-10 microcomputer control electronic universal testing machine is used in the quasi-static loading test. The disc specimens are fixed in pre-flexion on the experimental platform with the vertebrae, then the compressive loading is applied to the flexed discs (Fig. 1b). Before the experiment, the specimens were preloaded 3–5 times, and more pretreatments may also restore more physiological initial conditions. At the same time, it was found during the experiment that after 3–5 preloading, the stress–strain curves of the samples were close to overlapping compared to the previous one [23, 24].

The test samples are divided into 5 groups, the test samples were divided into 5 groups, each containing all 6 segments of the spine:

#### Healthy group

Healthy disc samples are loaded at quasi-static compressive strain rates of 0.0008/s, 0.008/s, 0.08/s, and high loading rate compressive strain rates of 0.2/s and 0.4/s.

#### Damaged group

**Fig. 2:**
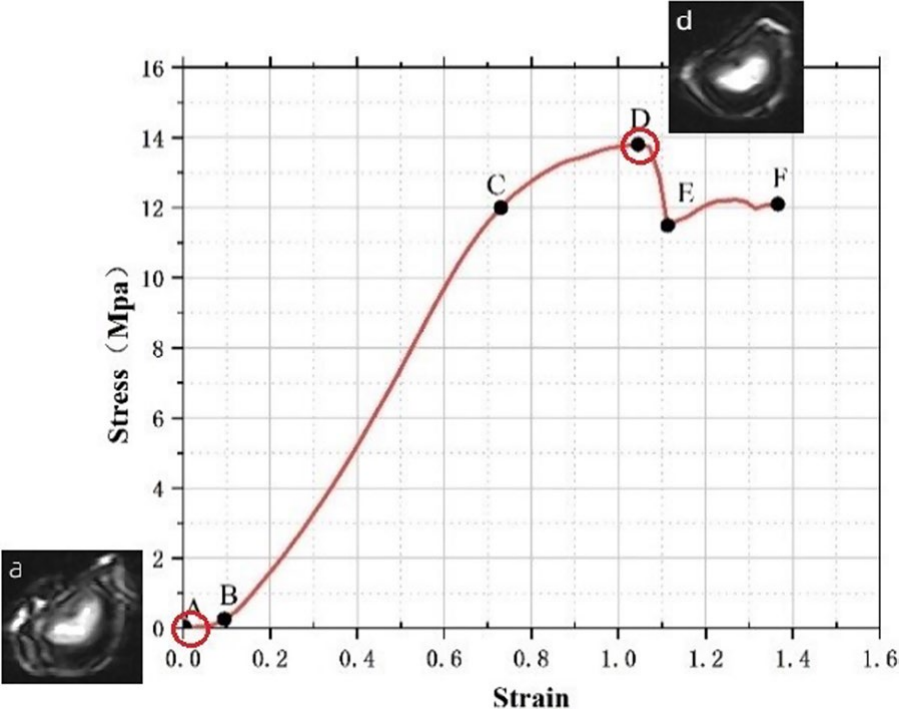
Description of stress–strain curve

Damaged disc is loaded adopting the same test method as the healthy group. In Fig. 2, point *C* represents the point of elastic limit, while point *D* represents the point of ultimate strength. Damaged samples are made from healthy sheep lumbar motion segments by flexing 5° and compressing up to point of ultimate strength (point *D*) at strain rate of 0.008/s to make damaged samples.

#### Magnetic resonance imaging (MRI) group

Siemens 1.5T MRI equipment is used to scan the healthy IVD and damaged IVD at quasi-static and high loading rate, respectively. And the scanning magnetic field is 1.5T, the interlayer spacing is 10% in all cases, the horizontal layer thickness is 4 mm, and the number of layers is 3. The sagittal plane layer thickness is 4 mm, and the number of layers is 11.

#### Internal displacement testing group

Quasi-static compressive strain rate is 0.008/s, high loading rate compressive strain rate is 0.2/s, and stop displacement is 2 mm.

#### Constitutive equation fitting group

Quasi-static compressive strain rates are 0.0008/s, 0.008/s, 0.08/s, high loading rate compressive strain rates are 0.2/s, 0.4/s, and the stop displacement is 2mm.

### Description of stress–strain curves

According to the figure, under the pre-flexion compression, there are five stages in the stress–strain curve of the disc: toe stage (AB), elastic stage (BC), strengthening stage (CD), collapse stage (DE), and stress plateau stage (EF) (Fig. 2). The samples failure during collapse stage, while undergo continuous and stable collapse during stress plateau stage.

### Internal displacement for measurement

**Fig. 3:**
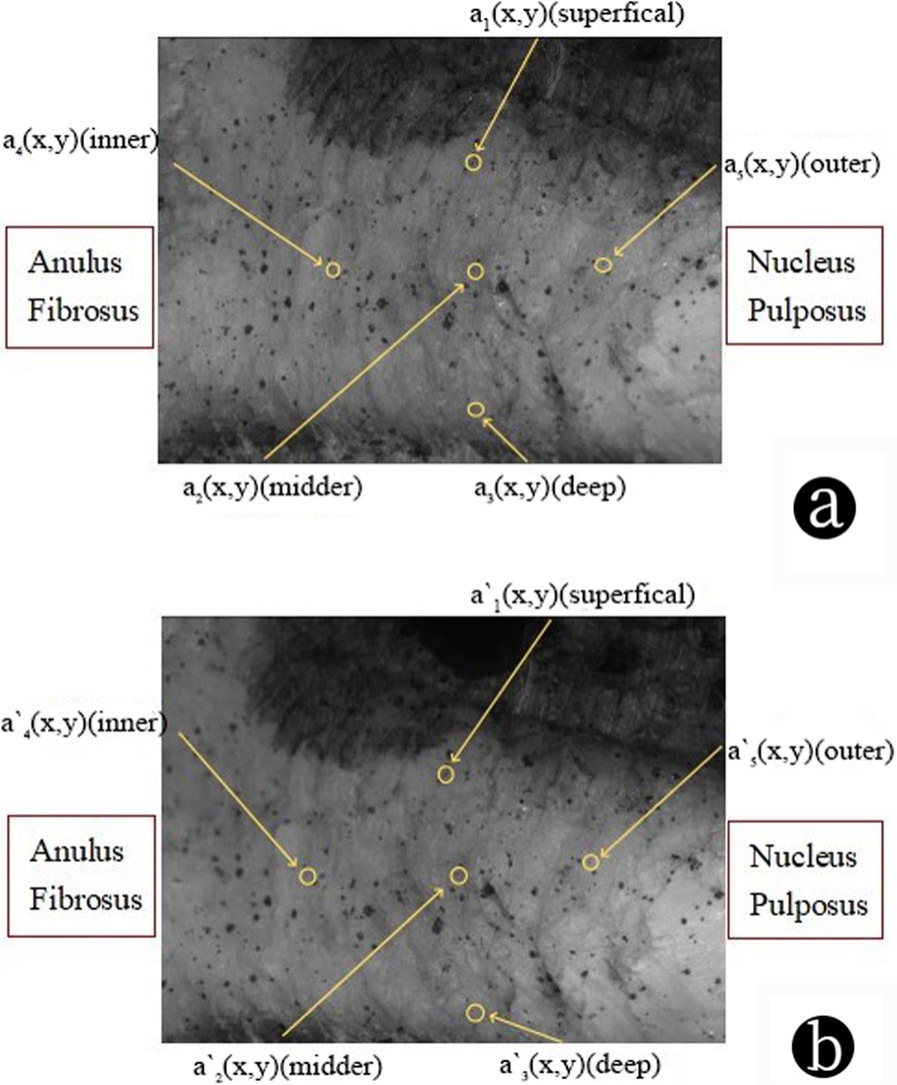
AF image of disc collected by DIC technology. The black marks are iron oxide nanoparticles. **a** The collected images before loading; **b** The collected images after loading

In the internal displacement experiment, the tracking marker points of DIC technology are used to calculate the strain magnitude of different regions before and after intervertebral disc loading. Figure 3 shows the fiber ring images of the intervertebral disc before and after loading. The black spots on the surface of the fiber ring are iron oxide nanoparticles. A set of marker points *a*_1_, *a*_2_, and *a*_3_ with similar *x* values were selected, and the changes in y values before and after loading at the selected midpoint were calculated and compared to obtain the values of axial displacement. We selected a set of marker points *a*_4_, *a*_2_, and *a*_5_ with similar y values, calculated and compared the *x* value changes before and after loading at the selected midpoint, and obtained the radial displacement values.

### Rate-dependent constitutive relationship of the disc

**Fig. 4:**
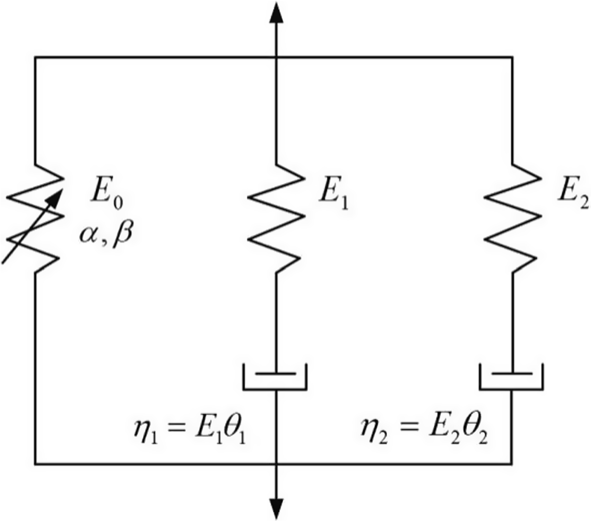
ZWT nonlinear viscoelastic model

Figure 4 is the schematic of the ZWT nonlinear viscoelastic constitutive model, while the first Maxwell element describes the viscoelastic response under quasi-static loading, and the second Maxwell element describes the viscoelastic response at high strain rate.

In the case of constant strain rate loading, the stress formula for the ZWT model is:

1 $$\sigma \left( t \right) =\, E_{0} \varepsilon \left( t \right) + \alpha \varepsilon \left( t \right)^{2} + \beta \varepsilon \left( t \right)^{3} + E_{1} \theta_{1} \dot{\varepsilon }\left( t \right)\left[ {1 - \exp \left( { - \frac{\varepsilon }{{\dot{\varepsilon }\left( t \right)\theta_{1} }}} \right)} \right] + E_{2} \theta_{2} \dot{\varepsilon }\left( t \right)\left[ {1 - \exp \left( { - \frac{\varepsilon }{{\dot{\varepsilon }\left( t \right)\theta_{2} }}} \right)} \right]$$

At low strain rate, it means that the high strain rate response term begins to relax at the beginning of loading. Therefore, the high strain rate integration term is negligible. Then Eq. (1) can be simplified to:

2 $$\sigma = E_{0} \varepsilon + \alpha \varepsilon^{2} + \beta \varepsilon^{3} + E_{1} \theta_{1} \dot{\varepsilon }\left[ {1 - \exp \left( { - \frac{\varepsilon }{{\dot{\varepsilon }\theta_{1} }}} \right)} \right]$$

At high strain rates, the impact test time is very short, and the low-frequency response does not have time to relax, so Maxwell *I* can be treated as a spring, and the elastic modulus is *E*_1_. Then Eq. (1) can be simplified to:

3 $$\sigma = \left( {E_{0} + E_{1} } \right)\varepsilon + \alpha \varepsilon^{2} + \beta \varepsilon^{3} + E_{2} \theta_{2} \dot{\varepsilon }\left[ {1 - \exp \left( { - \frac{\varepsilon }{{\dot{\varepsilon }\theta_{2} }}} \right)} \right]$$

For the parameter fitting of the ZWT equation, a total of five rates are tested, while three of them are used for fitting and the other two rates are used for validation. That is, the rate used for validation is different from the rate used for fitting. In addition, in order to ensure the accuracy of parameter fitting, five lumbar are tested, and each lumbar is divided into five segments for testing.

The flowchart of the fitting process is as follows:

1. The stress–strain results of quasi-static strain rates of 0.0008/s and 0.008/s obtained from experiments are substituted into $E_{1} \theta_{1} \dot{\varepsilon }\left[ {1 - \exp \left( { - \frac{\varepsilon }{{\dot{\varepsilon }\theta_{1} }}} \right)} \right]$ to get the parameters *E*_1_ and *θ*_1_
2. Substitute *E*_1_ and *θ*_1_ into Eq. (2), and get *E*_0,_ *α* and *β* by using the stress–strain results of quasi-static strain rate 0.008/s obtained in the experiment
3. Substitute the obtained *E*_0,_ *α*, *β*, *E*_1_ and *θ*_1_ into Eq. (3), and get *E*_2_ and *θ*_2_ through the stress–strain results of high strain rate 0.2/s obtained by experiment
4. By comparing the stress–strain curve obtained by the experiment at the strain rate 0.08/s with the stress–strain curve obtained by the fitting equation, the accuracy of the quasi-static fitting equation can be made sure
5. Finally, by comparing the stress–strain curve obtained by the experiment at the strain rate 0.4/s with the stress–strain curve obtained by the fitting equation, the accuracy of the high rates of loading fitting equation can be made sure

### Statistical analysis

The difference between the mechanical performance parameters and the experimental measurements of internal displacement obtained from multiple compression tests on different segments of the same spine at the same rate is less than 5% (*p* < 0.05). The data points in the graph represent the average value.

## Result

### Mechanical behavior of lumbar disc failure

**Fig. 5:**
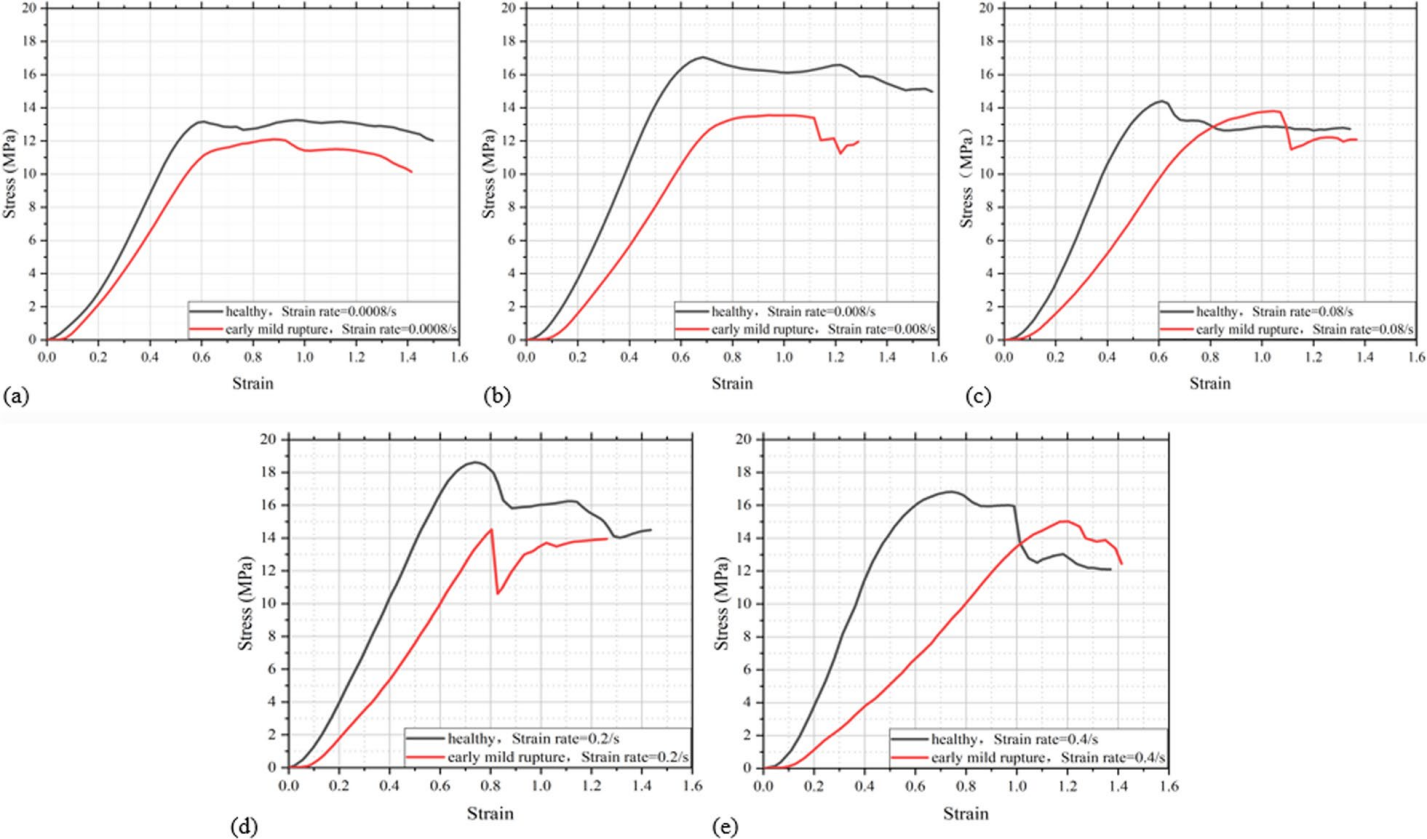
Stress–strain characteristic curve of the disc at different strain rates. quasi-static compression: **a** 0.0008/s strain rate, **b** 0.008/s strain rate, **c** 0.08/s strain rate; high rates of loading; **d** 0.2/s strain rate, **e** 0.4/s strain rate

Figure 5 shows the stress–strain curves of the disc samples at quasi-static and high rates of loading until failure.

#### The effect of damage

For healthy lumbar discs, under quasi-static compression, collapse stage and stress plateau stage do not appear in the stress–strain curve; under high rate of loading compression, the stress–strain all have collapse stage and stress plateau stage. However, for damaged disc, except at strain rate of 0.0008/s, collapse stage and stress plateau stage appear in all other strain rates.

In addition, the elastic modulus of damaged disc is significantly lower than that of healthy discs, and the difference between the two grows with increasing strain rate. Additionally, the toe stage of the failure disc is longer than that of a healthy disc, and the toe stage becomes longer with increasing strain rate (Table 1).

**Table 1:** The length of the toe stage after damage and the percentage of elastic modulus that varies with increasing strain rate

Strain rate	Rate of change in elastic modulus (%)	The rate of change in relative length of the toe stage (%)
0.0008	25.61	60.59
0.008	35.98	49.88
0.08	33.78	26.15
0.2	29.33	76.32
0.4	57.10	25.34

#### Influence of strain rate

For healthy discs, the stress–strain curves perform a similar multi-segment characteristic without a sharp drop under quasi-static compression. And under high rate of loading compression, with a sound of cracking, the stress–strain curves plunge, showing a similar multi-segment characteristic.

For damaged discs, at low strain rate, the strengthening stage of curve is longer and smoother, indicating a stronger resistance to failure. However, as the strain rate increases, the strengthening stage becomes shorter, with a significant decrease in the resistance to failure.

Based on the stress–strain curve in Fig. 5, the elastic limit, ultimate strength, and elastic modulus (Table 2) of both healthy and damaged lumbar discs at different strain rates can be acquired by calculation. From Table 2, the following conclusions can be drawn:

**Table 2:** Mechanical performance parameters of the disc at different strain rates

Sample	Strain rate	Elastic limit (MPa)	Ultimate strength (MPa)	Elastic modulus (MPa)	The relative length of the toe stage (%)
Healthy lumbar disc	0.0008	10.63	13.17	32.80	6.47
	0.008	13.64	17.05	37.80	8.12
	0.08	12.00	14.4	37.00	11.05
	0.2	16.92	18.61	31.70	8.36
	0.4	14.70	16.81	39.63	10.22
Early mild failured lumbar disc	0.0008	10.39	12.1	24.40	10.39
	0.008	11.87	13.55	24.20	12.17
	0.08	10.85	13.79	21.50	13.94
	0.2	13.87	14.52	22.40	14.74
	0.4	13.40	15	17.00	12.79

#### The effect of damage

The elastic modulus, elastic limit, and ultimate strength of damaged lumbar discs are significantly reduced, indicating a decrease in stiffness and strength of the discs after failure. This makes the damaged lumbar discs more susceptible to deform under compression and less likely to recover to their original state.

#### The effect of strain rates

With the growth of strain rate, both the elastic limit and ultimate strength of healthy and damaged discs show an increasing trend. With increasing strain rate, the elastic modulus of healthy discs increases obviously, while the elastic modulus of the damaged samples decreases obviously.

### The results of MRI for lumbar discs

**Fig. 6:**
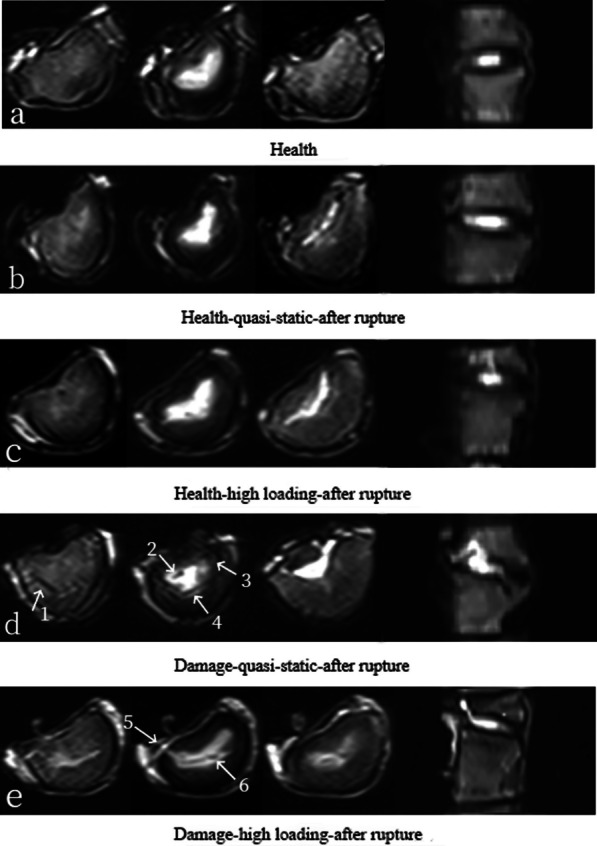
MRI pictures of different moving segments

By comparing Fig. 6b, d, it can be seen that the healthy lumbar disc does not show obvious cracks when loaded at quasi-static rate to failure, while the upper endplate (arrow 1) and AF (arrows 2–4) of the damaged lumbar disc has obvious cracks.

Comparing Fig. 6c, e, it can be seen that the healthy lumbar disc does not show obvious signs of damage when loaded to failure at high rate, while the damaged lumbar disc has a serious damage to the NP prolapse (arrows 5–6).

Comparing Fig. 6d, e, it can be seen that the herniation occurs in the junction between the posterolateral annulus and the upper endplate, when damaged lumbar discs are loaded to failure both at quasi-quiescent and high loading rates.

### Internal displacement distribution of lumbar disc

**Fig. 7:**
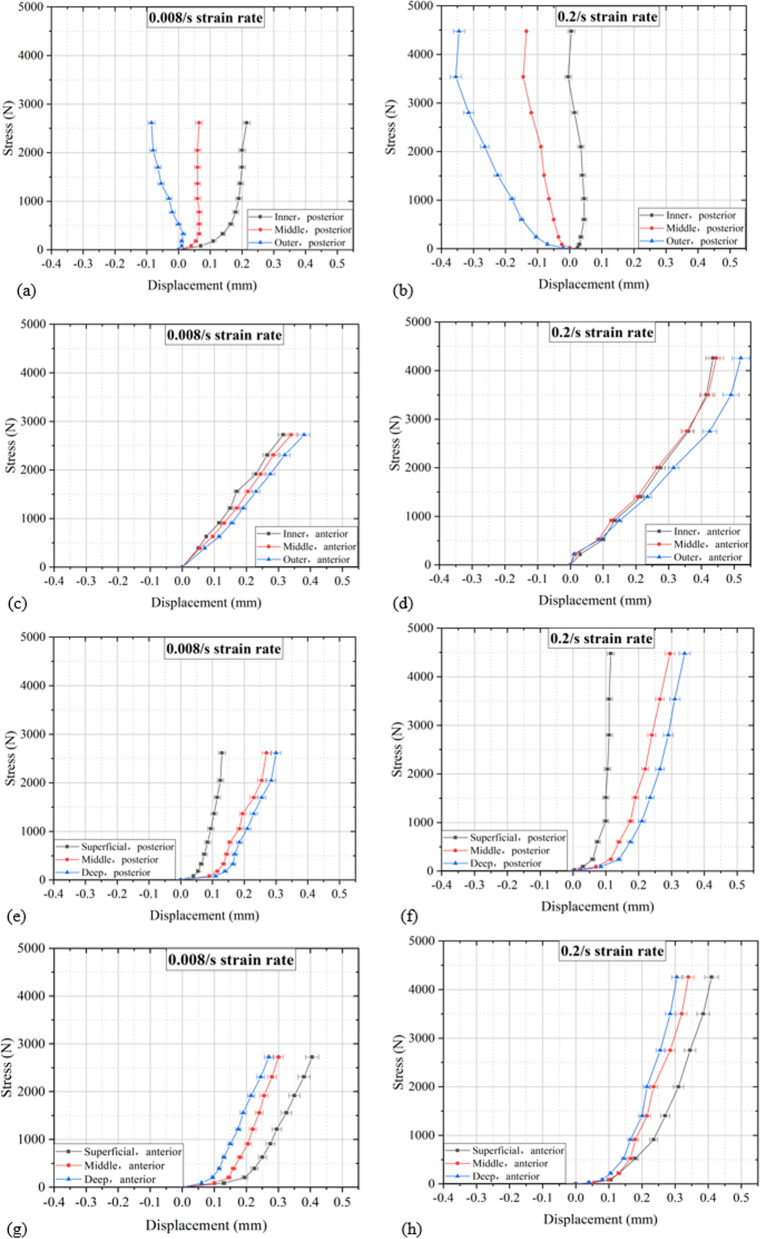
Radial displacement distribution of posterior AF. **a** 0.008/s radial displacement distribution, **b** 0.2/s radial displacement distribution; radial displacement distribution of anterior AF. **c** 0.008/s radial displacement distribution, **d** 0.2/s radial displacement distribution; axial displacement distribution of posterior AF. **e** 0.008/s radial displacement distribution, **f** 0.2/s radial displacement distribution. Axial displacement distribution of anterior AF. **g** 0.008/s axial displacement distribution, **h** 0.2/s axial displacement distribution

The internal displacement distribution of lumbar discs under flexion compression is shown in Fig. 7. The stopping displacement of flexion compression is approximately 1.6 mm for both quasi-static and high-rate loading. The stopping load under quasi-static loading is approximately 2670 N, and the stopping load under high-rate loading is approximately 4365 N.

Figure 7a, b shows the radial displacement distribution in posterior AF. It can be seen from the figure that under quasi-static loading, the inner AF has the largest displacement, the outer AF has the smallest displacement and AF moves away from the NP. After loading at high rate, the displacement of the outer AF is the largest, and the displacement of the inner AF is the smallest, and AF moves close to the NP.

Figure 7c, d shows the radial displacement distribution in anterior AF, while Fig. 7e–h shows the axial displacement distribution in posterior and anterior AF. According to the figures, the strain rate has little effect on the above displacement distribution.

### Rate-dependent constitutive relationship of the disc

**Fig. 8:**
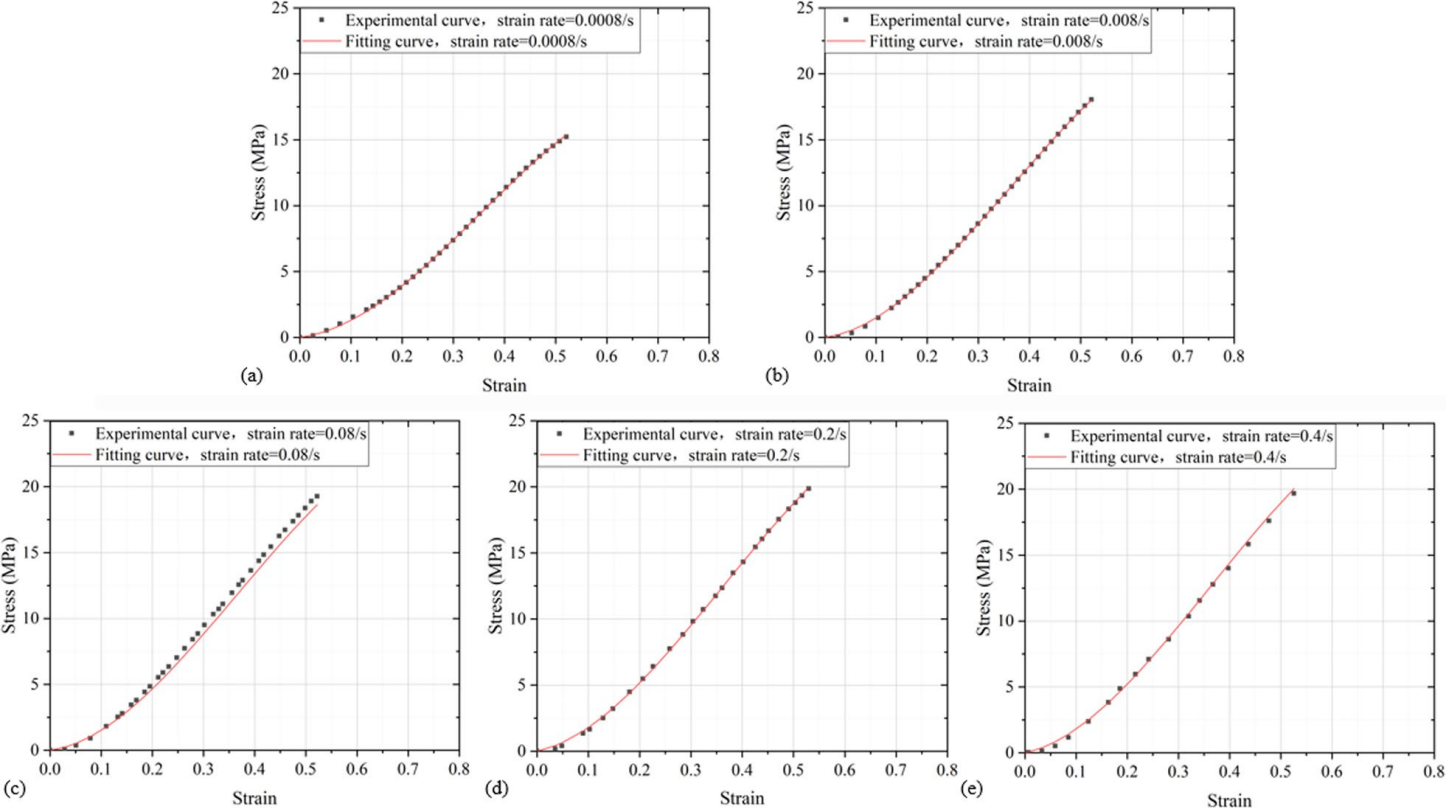
The stress–strain curves fitted at five different strain rates are compared with the experimental stress–strain curves: **a** 0.0008/s strain rate, **b** 0.008/s strain rate, **c** 0.08/s strain rate, **d** 0.2/s strain rate, **e** 0.4/s strain rate

Seven parameters are obtained by two sets of quasi-static experiments and one set of high loading experiments (Table 3), *E*_0_, *α*, *β* are the elastic constants of nonlinear springs, *E*_1_ and *θ*_1_ are respectively the elastic modulus and relaxation time of the first Maxwell, *E*_2_ and *θ*_2_ are correspondingly the elastic modulus and relaxation time of the second Maxwell. Figure 8a–e is stress–strain curves fitted at five different strain rates were compared with the experimental stress–strain curves, because the parameters used in the fitting curve process are obtained by the experimental curve data of Fig. 8a, b, d, so in these three figures, the experimental curve and the fitting curve are basically consistent. Figure 8c, e compares the stress–strain curve obtained by fitting the constitutive equation with the experimental curve, and it is found that under quasi-static compression (Fig. 8c) and high loading rate compression (Fig. 8e), the overlap between the experimental curve and the fitting curve is better, which can better describe the mechanical properties of the lumbar IVD before the elastic limit point. In order to describe the relationship between the model parameters and the mechanical properties of the IVD, the modulus of elasticity is calculated based on the stress–strain curve of the loading process:

4 $$E\left( \varepsilon \right) = \frac{{\sigma \left( {\varepsilon^{\prime } } \right) - \sigma \left( {\varepsilon_{0} } \right)}}{{\varepsilon^{\prime } - \varepsilon_{0} }} \approx \frac{{\sigma \left( {\varepsilon^{\prime } } \right)}}{{\varepsilon^{\prime } }}$$

where ${\upsigma }\left( {\varepsilon_{0} } \right)$ and $\varepsilon_{0}$ are the stress and strain at the last point of the linear segment in the loading curve, respectively, ${\upsigma }\left( {\varepsilon^{\prime } } \right)$ and $\varepsilon^{\prime }$ are the stress and strain at the initial point of the linear segment in the loading curve, respectively.

**Table 3:** The fitting parameters at different strain rates are summarized in

*E*_0_ (MPa)	*E*_1_ (MPa)	*E*_2_ (MPa)	*α* (MPa)	*β* (MPa)	${\uptheta }_{1}$ (s)	${\uptheta }_{2}$ (μs)
3	9.15	1.72	116.80	110.30	197.35	0.53

At low strain rate, substitute Eq. (2) into Eq. (4), then the measured modulus of elasticity is:

5 $$E\left( \varepsilon \right) = \left[ {E_{0} + \alpha \varepsilon^{\prime } + \beta \varepsilon^{\prime 2} } \right] + \frac{{E_{1} \theta_{1} \dot{\varepsilon }}}{{\varepsilon^{\prime } }}\left[ {1 - \exp \left( { - \frac{{\varepsilon^{\prime } }}{{\dot{\varepsilon }\theta_{1} }}} \right)} \right]$$

At high strain rates, substitute Eq. (3) into Eq. (4), then the measured modulus of elasticity is:

6 $$E\left( \varepsilon \right) = \left[ {\left( {E_{0} + E_{1} } \right) + \alpha \varepsilon^{\prime } + \beta \varepsilon^{{\prime}{2}} } \right] + \frac{{E_{2} \theta_{2} \dot{\varepsilon }}}{{\varepsilon^{\prime}}}\left[ {1 - \exp \left( { - \frac{{\varepsilon^{\prime } }}{{\dot{\varepsilon }\theta_{2} }}} \right)} \right]$$

In Eqs. (5) and (6), the first term is the elastic solid part of a viscoelastic solid, which is independent of the strain rate; while the second term is the fluid part of a viscoelastic solid, which is dependent on the strain rate. In addition, the second term is a strictly increasing function of the strain rate. In order to further analyze the effect of strain rate on elastic modulus, two extreme cases are considered:

In the first case when the strain rate is 0, the elastic modulus can be obtained from Eq. (5):

7 $$\begin{aligned} \mathop {\lim }\limits_{{\dot{\varepsilon } \to 0}} E\left( \varepsilon \right) & = \mathop {\lim }\limits_{{\dot{\varepsilon } \to 0}} \left\{ {\left[ {E_{0} + \alpha \varepsilon^{\prime } + \beta \varepsilon^{\prime 2} } \right] + \frac{{E_{1} \theta_{1} \dot{\varepsilon }}}{\varepsilon }\left[ {1 - \exp \left( { - \frac{{\varepsilon^{\prime } }}{{\dot{\varepsilon }\theta_{1} }}} \right)} \right]} \right\} \\ & \quad = E_{0} + \alpha \varepsilon^{\prime } + \beta \varepsilon^{\prime 2} \\ \end{aligned}$$

According to Eq. (7), the first part of the equation is a strain rate independent value of $E_{0} + \alpha \varepsilon^{\prime } + \beta \varepsilon^{\prime 2}$, while the second part is 0 when the strain rate is extremely low, indicating that the fluid part of the material has no contribution to the elastic modulus at this time.

In the second case when the strain rate is infinity, the elastic modulus can be obtained from Eq. (6):

8 $$\begin{aligned} \mathop {\lim }\limits_{{\dot{\varepsilon } \to \infty }} E\left( \varepsilon \right) & = \mathop {\lim }\limits_{{\dot{\varepsilon } \to \infty }} \left\{ \begin{gathered} \left[ {\left( {E_{0} + E_{1} } \right) + \alpha \varepsilon^{\prime } + \beta \varepsilon^{\prime 2} } \right] + \hfill \\ \frac{{E_{2} \theta_{2} \dot{\varepsilon }}}{\varepsilon }\left[ {1 - \exp \left( { - \frac{{\varepsilon^{\prime } }}{{\dot{\varepsilon }\theta_{2} }}} \right)} \right] \hfill \\ \end{gathered} \right\} \\ & \quad = \left( {E_{0} + E_{1} + E_{2} } \right) + \alpha \varepsilon^{\prime } + \beta \varepsilon^{\prime 2} \\ \end{aligned}$$

According to Eq. (8), the first part of the equation is a strain rate independent value of $\left( {E_{0} + E_{1} } \right) + \alpha \varepsilon^{\prime } + \beta \varepsilon^{\prime 2}$, while the second part is *E*_*2*_ when the strain rate is extremely high, indicating that the fluid part of the material has the greatest contribution to the elastic modulus at this time.

## Discussion

### Stress–strain curve of failure

The first finding is that the failure curves of healthy and damaged lumbar discs show different multi-segment characteristics. For healthy lumbar discs, only under high rates of compression, collapse appears in the stress–strain curve with a crack sound; for damaged lumbar discs, the stress–strain curve collapses regardless of whether it is under quasi-static or high-rate compression (Fig. 5). This is consistent with the results of the MRI examination (Fig. 6), which shows that there are no significant cracks when a healthy lumbar disc is loaded to failure, either at quasi-static rates or at high loading rates; when the damaged lumbar discs is loaded to failure, it is accompanied with obvious cracks.

The second finding is that the failure curve shape of the damaged lumbar disc is different at different strain rates: at low strain rate, its strengthening stage is longer and smoother, indicating that its ability to prevent the destruction of the specimen is stronger; as the strain rate increases, the strengthening stage becomes shorter and shorter, indicating that its ability to prevent the destruction of the specimen is significantly reduced (Fig. 5), which is consistent with the results of the MRI examination. MRI results show that the herniation occurs in the junction between the posterolateral annulus and the upper endplate when damaged lumbar discs are loaded to failure both at quasi-quiescent and high loading rates, which is consistent with the experimental results of Wade et al. [25, 26].

The third finding is that, for damaged lumbar discs, there is a large crack in the central area of the upper endplate under quasi-static loading to failure, and the junction of the NP and AF cracks intermittently (Fig. 6d); when damaged lumbar discs is loaded at a high rate to failure, severe damage to NP prolapse occurs (Fig. 6e). The above results show that the stress distribution inside the damaged lumbar disc changes as the strain rate increases [27–29]. The above results are consistent with the findings of Newell et al. [30–32], who finds that at high rate of loading, the NP does not affect the transfer of loads through or absorbed by the IVD, and at these rates, AF rather than NP may play the most important role in transferring the load and absorbing energy. In summary, the clinical failure of the lumbar disc is not only due to compression, but also the uneven distribution of stress in the lumbar disc. For healthy lumbar intervertebral discs, no protrusion of the nucleus pulposus occurred during quasi-static or high-speed loading until rupture (plateau stage on stress–strain curve); for early injury of lumbar intervertebral discs, both quasi-static and high-speed loading until rupture (plateau stage on stress–strain curve) result in nucleus pulposus prolapse. In addition, the stress at the strength limit of early damaged intervertebral discs is significantly lower than that at the strength limit of healthy intervertebral discs.

The above research results indicate that for the high-risk population of lumbar disc injury, such as drivers, nurses, and other people who bend and sit for a long time, as well as heavy manual workers such as heavy industry workers and farmers, it is necessary to avoid moving heavy objects quickly in daily life. Especially for individuals who have already suffered from lumbar disc injuries, it is important to avoid using unfavorable postures such as forward flexion and twisting to quickly move heavy objects, in order to prevent the injury from worsening and the occurrence of lumbar disc herniation.

### Internal displacement distribution

The fourth finding is that, with quasi-static loading, the AF moves away from the NP, and the inner AF is displacement maximum; at high rates of loading, the AF moves closer to the NP, and the outer AF is displacement maximum (Fig. 7). This is consistent with the MRI results [33]. During pre-flexion compression, the NP does not change its shape and position, while the anterior AF bulges and the posterior AF stretches. It is speculated that this may be the reason for the radial displacement of the posterior moving away from the NP under quasi-static loading and the action of hydrostatic pressure [34]. In addition, the stop displacement of flexion compression is approximately 1.6 mm, the stop load corresponding to quasi-static loading is approximately 2670 N, and the stop load corresponding to high-rate loading is approximately 4365 N. In summary, under quasi-static compression, the fibrous rings of the back and abdomen move away from the nucleus pulposus; at high loading rates, the AF in the back moves toward the nucleus pulposus, while the AF in the abdomen moves away from the nucleus pulposus, which may be the reason for the early injury of the nucleus pulposus to the back of the intervertebral disc (Fig. 6e). In addition, as the strain rate increases, the stiffness of the lumbar intervertebral disc significantly increases.

### Constitutive equation fitting

The fifth finding is that the ZWT nonlinear viscoelastic constitutive model was used to describe the mechanical behavior of lumbar discs during quasi-static and high-rate loading, and the fitting results were in good agreement with the experimental curve (Fig. 8). ZWT model is improved based on the standard linear solid model in the form of Maxwell, a nonlinear spring instead of its linear spring can effectively describe viscoelastic mechanics behavior of materials related to strain rate at different strain rate, studies have adopted the ZWT model description has been adopted to describe the mechanical properties of different materials under tensile and compressive loading, small and large deformation, quasi-static and high rates, the physical significance of the model parameters is still unclear.

## Limitation of the study

The damage model is only made by the pre-flexion loading, without considering the influence of the torsion loading; the influence of age and gender has not been considered, which have a great influence on the type of failure and protrusion.

## Conclusion

In summary, the research results indicate that compared with healthy IVD, the elastic modulus, elastic limit, and ultimate strength of injured lumbar IVD are significantly reduced, and the stress–strain curve of damaged specimens is more prone to collapse. Under quasi-static loading conditions, the strengthening stage of the IVD becomes shorter at high loading rates, and the resistance to failure is significantly reduced. In addition, as the strain rate increases, the elastic limit and ultimate strength of both healthy and damaged IVD show an increasing trend. The elastic modulus of healthy discs significantly increases, while the elastic modulus of damaged IVD significantly decreases. The high loading rate hardly affects the axial displacement of the fiber ring and the overall distribution of axial radial displacement in the forward bending state, but has a significant impact on the internal radial displacement distribution of the posterior fiber ring. MRI images indicate that injuries and high loading rates are more likely to cause disc herniation. The ZWT constitutive equation can well express the stress–strain relationship of IVD under high loading rates. Therefore, the research content of this article reflects the impact of injuries and high loading rates on IVD rupture in daily life, providing theoretical support for the prevention and treatment of LDH.

## Data Availability

The datasets used and/or analyzed during the current study are available from the corresponding author on reasonable request.

## Abbreviations

LDH: Lumbar disc herniation
IVD: Intervertebral disc
AF: Annulus fibrosus
NP: Nucleus pulposus
ZWT: Zhu–Wang–Tang
MRI: Magnetic resonance imaging
DIC: Digital image correlation
ANOVA: A one-way analysis of variance
EPDM: Ethylene propylene diene monomer
SHPB: Split Hopkinson pressure bar
